# Post-traumatic stress disorder interventions for children and adolescents affected by war in low- and middle-income countries in the Middle East: systematic review

**DOI:** 10.1192/bjo.2022.552

**Published:** 2022-08-08

**Authors:** Aseel F. Alzaghoul, Alison R. McKinlay, Marc Archer

**Affiliations:** Institute of Psychiatry, Psychology & Neuroscience, King's College London, UK; Centre for Mental Health and Wellbeing, Department of Psychology, Faculty of Behavioural Sciences, HELP University, Malaysia; Department of Behavioural Science and Health, Institute of Epidemiology and Health Care, University College London, UK

**Keywords:** Post-traumatic-stress disorder, psychological interventions, children and adolescents, low- and middle-income countries, Middle East

## Abstract

**Background:**

Millions of children and adolescents are exposed to wars, affecting their psychological well-being. This review focuses on psychosocial interventions in low and middle-income countries (LMICs) in the Middle East, where mental health services are limited.

**Aims:**

Our primary aim was to evaluate the effectiveness of trial-assessed psychosocial interventions in reducing post-traumatic stress disorder (PTSD) symptoms in children and adolescents aged ≤18 years who were exposed to war in LMICs in the Middle East. Changes in other psychological conditions and symptoms were evaluated where reported.

**Method:**

PubMed, Cochrane Library and Ovid were searched without year restriction, in December 2021. Previous review reference lists were also checked. Only studies published in English were included. Each study was evaluated for risk of bias and results are presented as a narrative synthesis.

**Results:**

Three group-based interventions were identified and evaluated across six studies: ‘Teaching Recovery Techniques’, ‘Writing for Recovery’ and ‘Advancing Adolescents’. Two studies took place in post-war settings, and four in a context of ongoing conflict. Positive experiences and improved social skills were indicated following most interventions, but Teaching Recovery Techniques was the only programme associated with a statistically significant reduction in PTSD score. Differences in follow-up interval limited comparability of outcomes.

**Conclusions:**

This review highlights a paucity of evidence for effective treatment options for children and adolescents affected by war from LMICs in the Middle East. Promising indications of reductions in PTSD symptoms, specifically from Teaching Recovery Techniques, require further rigorous evaluation and long-term follow-up.

War is associated with death, disability and invisible psychological injuries,^1^ many of which affect children and adolescents.^2^ Post-traumatic stress disorder (PTSD) among children affected by war is well documented,^3,4^ and although this may be linked to conflict and war settings,^5^ other factors can contribute to adverse mental health experiences, including poverty and deprivation.^6^ A total of 85% of the world's population live in one of the 153 countries currently classified as a low- and middle-income country (LMIC).^7^ This majority accounts for 80% of all people globally with mental health challenges;^8,9^ however, data on mental health prevalence and outcomes in these settings are lacking. Many children and adolescents affected by war live in low-resource countries, where mental health services are unavailable or inaccessible.^10^ Our review, therefore, focuses on psychological support for children and adolescents with PTSD in LMICs in the Middle East.

Many Middle Eastern countries, regardless of income class, have an insufficient number of psychiatrists to cope with demand, and lack specialised child and adolescent psychiatry (CAP) services.^11^ In a study by Clausen et al of 15 Middle Eastern countries (including Palestine and Jordan), the authors identified an almost complete absence of CAP services, resulting in a lack of established national training guidelines. Consequently, many young people are at risk of developing long-term mental health conditions that will go unidentified and untreated from an early age.^11^ The lack of CAP services is a global issue; however, research has shown that the demand is greater in low-resource countries not only because they lack essential resources, but also because 90% of children and adolescents live in LMICs whereas 95% of psychiatrists are in high-income countries.^11^ This makes the low-resource requirements and scalability of some psychosocial interventions particularly suited to LMICs in the Middle East (Iran, Iraq, Jordan, Lebanon, Palestine, Syria, Yemen and Turkey^7,12^).

Non-governmental organisations play an important role in providing mental health services for children in LMIC in the Middle East^10^ and assessing psychosocial concerns.^13^ Non-governmental organisations and academic divisions that specialise in child mental health have highlighted the need for mental health programmes and the scaling up of mental health services.^14^ The World Health Organization responded to this lack of services by developing the Mental Health Gap Action Programme, where psychological and psychosocial interventions are considered first-line therapy, including cognitive–behavioural therapy (CBT) and narrative exposure therapy.^5^

War has affected several Middle Eastern countries such as Palestine, Iraq and Lebanon for decades, whereas countries such as Syria have experienced intensive war more recently. A wave of protests and overthrowing of dictatorships across the region in 2010, known as the Arab Spring, led to intense violence in countries like Syria, with many people becoming internally displaced and/or refugees.^15^ Publications from the Middle East are scarce, particularly in CAP services;^11^ however, some evidence suggests a high prevalence of PTSD in countries such as Palestine and Iraq, compared with high-income, non-Middle Eastern countries.^16^ Dimitry's systematic review of 21 studies involving 11 000 children and adolescents in Palestine found PTSD prevalence to range from 23 to 70%.^17^ The percentage of Lebanese children and adolescents exposed to different traumatic experiences in Dimitry's review was also high (up to 94%).^17^ In another study, 43% of Lebanese children met PTSD criteria for war-related trauma that was experienced up to 10 years previously, highlighting a complex and potentially delayed response in processing trauma.^18^ One study of Iraqi children found that 14% had a diagnosis of PTSD,^19^ whereas 35–45% of Syrian children in Syria and Turkey are estimated to be experiencing PTSD symptoms.^20,21^ Given this high prevalence of PTSD and trauma, ongoing conflict in these war-affected regions and lack of services in the Middle East, the provision of effective interventions for this vulnerable population and evidence to guide future research are warranted and urgently needed.

## Rationale for the review

Before undertaking this review, we identified five other reviews^5,8,22–24^ that examined psychological/psychosocial interventions in LMICs; two reviews found suggestive evidence of efficacy of interventions targeting children;^22,23^ two other studies identified beneficial effects for trauma-focused interventions only or so-called first-line strategies, such as CBT;^8,24^ and one review found very low-quality evidence for children's psychological/psychosocial interventions.^5^ However, some of these reviews included studies with adults and children in the same sample, with more focus on results from adults in the sample,^5,23^ or they included studies with different designs other than randomised controlled trials (RCTs)^8^ or that were conducted in various humanitarian crisis settings such as natural disasters, mass violence and armed conflicts,^5,22^ rather than war and conflict specifically. There are no known recent reviews to our knowledge that focus on the unique context specific to LMICs in the Middle East, to summarise evidence on psychosocial intervention for war-affected children in this area.

Although recent reviews have evaluated PTSD interventions in LMICs more broadly,^5,8,22–24^ these have not focused solely on the Middle East, where armed conflicts and wars are currently ongoing. War-related stressors and trauma are expected to lead to heightened prevalence of mental health problems, particularly PTSD.^5^ There is good initial evidence that early interventions for children and adolescents can prevent long-term psychological problems such as PTSD;^6^ however, results require further investigation, using robust methods. A previous systematic review mentioned the lack of RCTs in LMICs;^22^ consequently, further research in this field could provide much needed guidance for future work and recommendations.

The aim of this systematic review is therefore to evaluate the effectiveness of available RCT-assessed interventions for children and adolescents who have been exposed to war and conflict in LMICs in the Middle East. Many children and adolescents previously living in LMICs in the Middle East have been displaced, with large populations moving into Middle Eastern countries not currently experiencing war, such as Jordan, Lebanon and Turkey.^25^ We therefore expanded our review focus to include children and adolescents who have been displaced to these regions. Four research questions are addressed: What are the available RCT-assessed interventions for children and adolescents? Are these interventions effective in reducing PTSD symptoms as a main outcome, and other psychological outcomes if measured, compared with control groups? Do children and adolescents benefit in different ways from the intervention based on demographic characteristics and war exposure? Finally, are there additionally reported benefits or consequences associated with these interventions (such as reduced distress, relief or new skill development)?

## Method

The Preferred Reporting Items for Systematic Reviews and Meta-analyses (PRISMA) guidelines were followed^26^ and the systematic review protocol was registered on the PROSPERO database (reference: CRD42019140370). Meta-analysis was considered but, because of the small number of studies and varied treatment implementation and assessment, qualitative narrative synthesis was used to summarise findings.

### Inclusion criteria

Studies were included if they were published in English in a peer-reviewed journal; were RCTs of psychological or psychosocial interventions; and were undertaken with children and adolescents aged ≤18 years, who are refugees or internally displaced, or have been exposed to wars and conflicts, and live in LMICs in the Middle East. All study participants indicated PTSD symptom scores above the clinical cut-off.

It is important to mention that although in the PROSPERO record we indicated the target age group to be <18 years, because of the lack of RCTs in this range, we included one study with participants aged ≤18 years. The average age of the study^27^ sample (range 12–18 years, average 14.25 years) was comparable with other included studies.

### Exclusion criteria

Studies were excluded if they were undertaken with children and adolescents who were refugees or internally displaced living in high-income countries (whether inside or outside the Middle East), conducted in LMICs outside the Middle East, did not measure PTSD as a primary or secondary outcome, or included a mixed age sample (i.e. adults and children) without results presented separately by age. In addition, studies were excluded when war or conflict was not identified as one of the causes of PTSD.

### Search strategy and selection process

The following databases were searched from their inception until December 2021: PubMed, Cochrane Library and Ovid (Medline, PsycINFO, Global Health and EMBASE). The following keywords were used: Effect*, treatment, intervention, ‘psychological interventions’, PTSD, trauma, ‘post trauma’, ‘posttraumatic stress disorder’, child, ‘school age’, adolescent, youth, ‘middle east’, ‘Arab countries’, ‘middle income’, ‘low income’, LMIC, war, conflicts, ‘armed conflicts’, refugee, ‘internally displaced’. Search results were filtered to include only RCT studies. Reference lists of previous systematic reviews^5,8,22,28,29^ were manually checked and suitable studies included. Titles and abstracts were reviewed independently against the inclusion/exclusion criteria by two authors (A.F.A., A.R.M.) and duplicates were removed. Full texts of the remaining articles were then assessed for inclusion by two authors.

### Data extraction

Author A.F.A. extracted the following data from all studies: country/location of the intervention, participant characteristics, type of intervention and control conditions, outcome measures, PTSD diagnosis or symptoms score at baseline/follow-up, number and duration of follow-up, subgroup analysis, war stressors, treatment fidelity, therapist training and any other important notes. The data used was that for participants who had completed follow-up.

### Quality assessment

We used the Cochrane Collaboration Risk of Bias Tool (CCRBT)^30^ to assess four main types of bias: selection bias, attrition bias, detection bias and reporting bias. Given the nature of psychological interventions, where participant blinding to treatment condition was not possible, the ‘blinding’ component of the CCRBT was not used. Additional risk of biases was assessed from miscellaneous sources. Assessments were conducted independently by two authors (A.F.A. and A.R.M.), using the grading system (high, low or unclear risk), with any disagreements resolved through discussion.

## Results

**Fig. 1:**
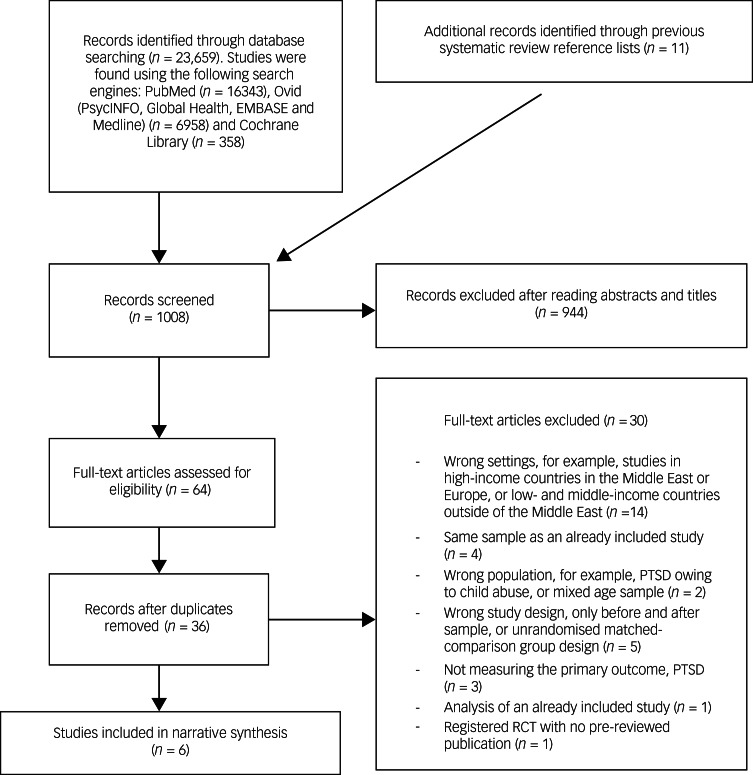
Flow diagram. PTSD, post-traumatic stress disorder; RCT, randomised controlled trial.

The systematic search yielded 1008 potentially relevant articles. Inspection of titles and abstracts reduced this to 64 articles, of which 28 were duplications. After full-text examination of the remaining 36 articles, six met inclusion/exclusion criteria, and data were then extracted (Fig. 1, Table 1).

**Table 1:** Characteristics of included studies

Study	Country/ location	Participant characteristics	Intervention type/implementation setting	Control	Therapist background/training and supervision	Measures	War stressor and exposure (across intervention and control groups)	Main results
Lange-Nielsen et al, 2012^31^	Gaza, Palestine	*N* = 124 Age = 12–17 (average 14.57) years Gender: 50% boys 50% girls	WfR school library, *n* = 66	Waitlist, *n* = 58	Professional psychologists from CFTCC. Trained for 1 day in administering WfR manual. Videotaped intervention sessions	1) Gaza Traumatic Event Checklist 2) CRIES 3) Revised Children Manifest Anxiety Scale 4) DSRS	Seeing mutilated body on television (intervention: 92.4%, control: 91.1%). Hearing shelling of the area (intervention: 86.4%, control: 91.1%)	The intervention did not decrease measured symptoms and there was an elevation in depressive symptoms following intervention
Barron et al, 2013^32^	Nablus, Palestine	*N* = 133 Age = 11–14 (average 11.08) years Gender: 73 boys 60 girls	School-based TRT *n* = 83	Waitlist *n* = 50	Twenty school counsellors were trained in TRT for 3 days by expert trainees from Children and War foundation. Two counsellors implemented the intervention, one for presenting and the other for observing	1) CRIES 2) DSRS 3) Traumatic Grief Inventory for Children 4) Impact on School Performance Scale 5) SDQ	Intervention: experiencing close shelling 79%, seeing a dead body 78.3%, family member injured 77.1% and seeing someone killed 74%. Control: seeing sexual assault 100%, witnessing torture 92%, physical confinement 84% and seeing a dead body 84%	PTSD, grief and depression decreased significantly in intervention group post-intervention
Barron et al, 2016^33^	Palestine	*N* = 139 Age = 11–15 (average 13.5) years Gender: 56 boys 83 girls	School-based TRT *n* = 75	Waitlist *n* = 64	School counsellors were trained in TRT for 3 days by expert trainee from Children and War foundation. Two counsellors or a small group implemented the intervention, one for presenting and the other/s for observing	1) Exposure to War Stressor Questionnaire 2) CRIES 3) DSRS 4) Adolescent Dissociative Experience Scale	Exposure across both groups: parents separated from each other 97.1%, used as human shield 96.4%, separated from family 95.6%, shot at by a sniper 94.2%, a member of the family killed 94.2%	Intervention led to less PTSD symptoms. Depression and dissociation remained the same for intervention group and increased in waitlist control group
Panter-Brick et al 2018^27^	Jordan	*N* = 176 (Syrian = 102) Age = 12–18 (average 14.25) years Gender: approximately 43 girls 59 boys (numbers of girls and boys were calculated using the percent of each gender)	Advancing Adolescents implemented in local urban centres *n* = 48	Waitlist *n* = 54	Mercy Corps NGO trained adults from local communities to enhance safety and psychosocial support. Sessions were monitored	1) Human insecurity and Human Stress Scale 2) SDQ 3) Arab Youth Mental Health 4) CRIES 5) Harvard Trauma Questionnaire and Gaza Checklist	Exposure across both groups: witnessing bombardment 80.7%, having their home searched by militia 71.5%, having seen homes demolished 54.9%, seen wounded/ dead bodies 53.8%	There was no programme effect on prosocial behaviour and PTSD. However, it improved the psychosocial well-being for both Syrian refugees and the Jordanian host community
Qouta et al, 2012^34^	Gaza, Palestine	*N* = 482 Age = 10–13 (average 11.29) years Gender: 49.4% girls 50.6% boys	School-based TRT *n* = 242	Waitlist *n* = 240	Four psychologists. Counsellors were trained by the first author.Weekly supervisory meetings held with the first author	1) Peritraumatic Dissociative Experience Questionnaire 2) CRIES 3) DSRS 4) SDQ	Not reported	The intervention had gender-related and risk-specific effectiveness
El-Khani et al, 2021^35^	Beqaa Valley, Lebanon	*N* = 119 Age = 9–12 years (average not mentioned) Gender: Only parents’ gender mentioned	TRT *n* = 41 TRT + parenting *n* = 38	Waitlist *n* = 40	Teachers. First author trained teachers on TRT remotely and she is an approved TRT trainer/ parenting intervention was a pre-recorded video material of 8 h split across 2 days. Videotaped intervention sessions	Children: 1) CRIES 2) SCARED 3) DSRS 4) SDQ Parents: 1) The Parenting Scale 2) IES-R 3) DASS 4) Family Background Questionnaire	The exposure was only decided through CRIES score as it is designed for war-affected children	All the scales showed best improvement in the TRT + parenting group. The enhancement was for both parents and children

WfR, Writing for Recovery; CFTCC, Child and Family Training and Counseling Center; CRIES, Child Revised Impact of Events Scale; DSRS, Depression Self-Rating Scale for Children; TRT, Teaching Recovery Techniques; SDQ, Strengths and Difficulties Questionnaire; PTSD, post-traumatic stress disorder; NGO, non-governmental organization; SCARED, Screen for Childhood Anxiety-Related Disorders; IES-R, Impact of Events Scale Revised; DASS, Depression–Anxiety–Stress Scale.

### Study samples and settings

Four of the included studies took place in Palestine with Palestinian children and adolescents in three different locations (Gaza, Nablus and West Bank),^31–34^ one took place in Jordan^27^ and involved Syrian refugees and Jordanian adolescents, and one took place in Lebanon^35^ and included Syrian refugees. Included participants in all studies were children and adolescents aged between 9 and 18 years, with average ages around 11 years in two of the studies in Palestine,^32,34^ 13 years in the third study in Palestine,^33^ and 14 years in the fourth study in Palestine and the study in Jordan.^27,31^ Gender distribution was almost equal in two studies,^31,34^ boys predominated (57.8% and 54.9%) in two studies^27,32^ and girls predominated (59.7%) in one study.^33^ The study by El-Khani et al^35^ did not report age and gender. This does not include all LMICs in the Middle East (Iran, Iraq, Jordan, Lebanon, Palestine, Syria, Yemen and Turkey^7,12^), and therefore results cannot be generalised to all Middle Eastern locations.

Gaza, Nablus and villages in the West Bank have been highly affected with ongoing military operations and violence. Two studies that took place in Gaza included schools in areas shelled during the Gaza war in 2008/2009^34^ and from a refugee camp in Gaza.^31^ Nablus and some villages near East Jerusalem in West Bank were selected in the studies by Barron et al^32,33^ because of high levels of ongoing military violence. Refugees displaced from Syria for an average of 2.7 years were included in the Jordan study^27^ as they experienced war and traumatic events related to war, such as witnessing bombardment before moving to Jordan. Syrian child refugees who were displaced to Lebanon and scored 17 or more on the Child Revised Impact of Events Scale (CRIES-13) were included in the Lebanon study.^35^

The combined sample size was 1099 participants, with sample sizes in contributing studies ranging from 102^27^ to 482.^34^ In the study by Panter-Brick et al,^27^ only Syrian children (56.3% and 60% in intervention and control groups, respectively) were included because Jordanian children were not exposed to war and their PTSD symptoms resulted from other causes, such as domestic abuse. The number of Syrian children (*n* = 102 (43 females); 48 in the intervention and 54 in the control conditions) was calculated from data provided in the paper. It was not possible to extract some data of interest separately for Syrian children as they were reported with Jordanian children as one group.

### War and trauma exposure

There were no significant differences between intervention and control groups on war and trauma exposure in any of the four studies that included these data (Table 1). One study with a ‘Teaching Recovery Techniques’ (TRT) intervention reported experienced traumatic events ranging from nine to 26 events per participant.^33^ Sixteen children in this study experienced 24 events each. The second TRT study reported that girls in the control group had higher levels of exposure compared with boys.^32^ All participants included in the Syrian sample at the baseline of the Panter-Brick et al study^27^ reported an average of 6.36 traumatic events, and 82.5% of Syrian refugees reported four or more lifetime trauma exposures.

The nature and types of traumatic events and stressors reported (Table 1) were war related. War events such as house or area shelling was reported in one TRT study^33^ (79% of intervention group reported this event), the ‘Writing for Recovery’ (WfR) study^31^ (86.4% in the intervention group and 91.1% in the control group) and the ‘Advancing Adolescents’ study^27^ (54.9%). Seeing a dead or injured body, whether a family member or a stranger, was also reported in two of the TRT studies (74–78%^32^ and 94%^33^), the WfR study (91–92%)^31^ and the Advancing Adolescents study (53.8%).^27^ All control group participants in one TRT study reported seeing someone sexually assaulted (100%).^32^

### Intervention and control types

All studies implemented school-based interventions except for the study by Panter-Brick et al,^27^ where the intervention took place at different community centres in Jordan. All interventions were implemented in groups rather one-to-one sessions. Three types of interventions were used: one CBT-oriented, one derived from narrative therapy (all trauma-informed) and one community-based psychosocial care intervention. These three are detailed below.

#### Teaching Recovery Techniques

TRT^32–35^ was developed by the Children and War Foundation to be used with children and adolescents in wars and disaster contexts.^33^ It is a skills-based programme, and derived from trauma-focused CBT,^35^ delivered by teachers or school counsellors, consisting of five sessions lasting for 90 min each. Sessions aim to help students understand trauma, cope with loss, and learn strategies to manage PTSD symptoms such as intrusive memories, hyperarousal and avoidance. School counsellors who delivered the programme first attended a 3-day training programme delivered by two specialists from the Children and War Foundation.

The study by El-Khani et al^35^ added three additional sessions with a parenting component (TRT + P) to one of the intervention groups. They identified parenting skills through systematic and analytical approaches, and they identified parental needs from examples mentioned when interviewing parents in conflicts areas. The new component aimed to understand behavioural change and to enhance positive child–parent interaction through simple techniques like rewarding desirable behaviour by using available material in low-resource sittings, such as behavioural charts.^35^ It is important to mention that this study^35^ was implemented during the COVID-19 pandemic, and that the intervention was implemented online.

#### Writing for Recovery

WfR^31^ is a manualised intervention based on narrative therapy techniques, also developed by the Children and War Foundation. The programme is for children and adolescents with a history of trauma, aged 12–18 years.^36^ It includes six writing sessions over three consecutive days: two sessions per day (15 min each), with a break of 10 min in-between. The writing tasks progress from general expression of emotions and detailing traumatic memory to developing insightful perspectives, with the intention to develop new narration for the traumatic event by facilitating and reframing insight. The intervention does not require specialist mental health training and was delivered by teachers after completing a 1-day training programme.

#### Advancing Adolescents

Advancing Adolescents^27^ is an 8-week programme of 16 sessions (two sessions per week), originally undertaken by Mercy Corps (an international non-governmental organisation). The programme is informed by the profound stress attunement framework, which focuses on psychosocial care for vulnerable children and adolescents and improving social interactions via a community-based, non-clinical setting. It consists of three main elements: safety, support and structured group activities for youths living in humanitarian crisis settings. Participants choose their preferred activity, such as playing sports, art and crafts, and vocational or technical skills activities. The programme was delivered by adult volunteers from the community who were trained by Mercy Corps.

All included studies compared a psychological or psychosocial intervention to a control group, except for the study by El-Khani et al,^35^ which compared an intervention group (TRT + P) with a comparison group (TRT only) and control group. Most studies used waitlist control.^27,32–35^ Authors of one study^31^ reported a deviation in the study protocol, as the control group received the WfR intervention after finishing only one of the two planned assessments. The reason this occurred was because of a ‘communication difficulty’.

### Measures of PTSD

The CRIES^37^ was used to assess PTSD symptoms in the six included studies (see Table 1).^27,31–35^ The CRIES is a self-report questionnaire for 8- to 18-year-olds, developed by the Children and War Foundation to assess risk of developing PTSD. The measure has eight-item (four for intrusion and four for avoidance) and 13-item (with an additional five items for arousal symptoms) versions.^38^ It has good face and construct validity, a stable factor structure and has been adapted to different languages and locations.^37^

Additional tools were used in two studies. Authors of the first study, evaluating Advancing Adolescents,^27^ used the Harvard Trauma Questionnaire,^39^ and authors of the second study, evaluating WfR, used the Gaza Traumatic Event Checklist,^40^ developed to assess trauma level (specifically for Palestinian children in Gaza), in addition to the CRIES measure.

### Other psychological outcomes measures

Depression was assessed in five studies^31–35^ with the Depression Self-Rating Scale for Children (DSRS).^41^ Peritraumatic dissociation was assessed in two studies^33,34^ with the Peritraumatic Dissociative Experience Scale.^42^ Anxiety was assessed in two studies^31,35^ with the Revised Children Manifest Anxiety Scale^43^ and the Screen for Childhood Anxiety-Related Disorders.^44^

The Strengths and Difficulties Questionnaire (SDQ)^45^ was used in four studies^32–35^ to assess psychological distress. The SDQ is a screening tool to measure behaviours and emotion in children. It can be completed independently by children and adolescents, if aged ≥11 years, or their teachers or parents. It contains 25 items (five scales with five items each): emotional symptoms subscale, conduct problems subscale, hyperactivity/inattention subscale, peer relationships problems subscale and prosocial behaviour subscale. Panter-Brick et al used the SDQ, in addition to the Human Insecurity and Human Distress Scale.^46,47^

#### War exposure measures

Although four studies included questions about war stressors and exposure, formal war exposure measures were used only in one study. Barron et al^33^ used the Exposure to War Stressor Questionnaire,^48^ which is a 26-item measure of ‘yes’ or ‘no’ questions and has no clinical cut-off.

#### Translation

Measures used in all included studies were in Arabic. All measures were already available in Arabic and validated in previous studies.

### Follow-up

Follow-up frequency ranged from one to three sessions after baseline assessment across the studies (see Table 2). Follow-up duration typically ranged from 2 weeks to 2 months post-intervention for the first follow-up. For the studies in which two follow-ups were completed, the second follow-up took place 12 weeks to 4/5 months post-intervention.

**Table 2:** Post-traumatic stress disorder symptoms by study and intervention/measure, with follow-up intervals

Study	Intervention/PTSD measure	Baseline (% above clinical cut-off, intervention/control)	Follow-up (% above clinical cut-off, intervention/control)						
			Immediately post-intervention	2 weeks	3 weeks	4 weeks	8 weeks	12 weeks	4–5 months
Lange-Nielsen et al, 2012^31^	WfR/CRIES	53%/60.3%	Time point 2 45.5%/ 56.9%		Results for both groups after the control group received the intervention were merged and called time point 3: 47.6%. Control group received intervention between time points 2 and 3				4 months post-intervention for control group. 5 months for intervention group. Merged results: time point 4: 48.8%
Qouta et al, 2012^34^	TRT/CRIES	64%/43%				64%/41%			
Barron et al, 2013^32^	TRT/CRIES^a^	63.9%/50%		33.7%^b^/44%					
Barron et al, 2016^33^	TRT/CRIES^a^	PTSD symptoms were the same in both groups, but no percentages were provided. Rather, the mean scores on the CRIES were 25.59 and 24.67 for intervention and control groups, respectively (these scores were considered above clinical cut-off)		59%^b^/87%					
Panter-Brick et al 2018^27^	Advancing Adolescents/CRIES		Not possible to extract information specific to target population						
El-Khani et al 2021^35^	TRT or TRT + parenting/CRIES	Time point 1					Time point 2	Time point 3	

PTSD, post-traumatic stress disorder; WfR, Writing for Recovery; CRIES, Child Revised Impact of Events Scale; TRT, Teaching Recovery Techniques.

a. CRIES: questionnaire was used one month before programme delivery. All other measures: 2 weeks before programme delivery.

b. Significant reduction in intervention condition.

### Outcomes

#### PTSD-related outcomes

Authors of three studies found that the TRT intervention was effective in decreasing PTSD symptom scores.^32,33,35^ Authors of the fourth TRT study^34^ found a gender-related and risk-specific effectiveness; girls with lower severity of peritraumatic dissociation at time point 2 and boys at time point 2 had reduced PTSD symptoms.^34^ However, a correction to results for this study indicated that these differences were not statistically significant.^49^ No treatment effect was found in studies implementing the Advancing Adolescents intervention or WfR programme.

##### Teaching Recovery Techniques

In one study by Barron et al,^32^ pre-intervention post-traumatic stress levels were significantly higher in the intervention group. Fifty-three students (63.9%) in the intervention group exceeded the diagnosis cut-off based on CRIES-8 scores, whereas 25 students (50%) had similar scores in the control group. After the intervention, 28 (33.7%) students in the intervention group and 22 (44%) students in the control group exceeded diagnosis cut-off.

In another study by Barron et al,^33^ pre-intervention post-traumatic stress levels were equal in the intervention and control groups. Average CRIES-8 scores were high in both groups (intervention: 25.59; control group: 24.67). A clinically significant difference was found at post-test in the intervention group, in which the mean decreased to 18.57 compared with 24.16 in control group. Twenty-nine (41%) students in the intervention group no longer met the diagnostic criteria for PTSD, compared with nine (13%) students in control group.

In the study by El-Khani et al,^35^ the three groups (TRT + P, TRT alone and waitlist) had the same score for avoidance and arousal symptoms at baseline; however, the TRT + P group had a higher intrusive symptoms score (TRT: *P* = 0.042; waitlist: *P* = 0.015). The TRT + P group demonstrated significant reductions in intrusive symptoms at time point 2 (*P* = 0.001) and time point 3 (*P* < 0.001), avoidance symptoms at time point 3 (*P* < 0.001) and arousal symptoms at time points 2 and 3 (all *P* < 0.001), compared with the waitlist group. The TRT group demonstrated significant reductions in intrusive and avoidance symptoms at time point 3 (*P* < 0.001 and *P* = 0.049), and arousal symptoms at time points 2 (*P* = 0.001) and 3 (*P* < 0.001), compared with the waitlist group. The TRT + P group showed significantly greater decreases in PTSD symptoms, particularly avoidance symptoms, than the TRT-only group.

In the study by Qouta et al,^34^ post-traumatic stress symptoms were markedly higher in the intervention group: 64% of students in the intervention group and 43% of students in the control group had clinically significant post-traumatic stress symptoms. At follow-up, this had increased to 45% in the control group and remained unchanged in the intervention group.

##### Writing for Recovery

In a study by Lange-Nielsen et al,^31^ baseline levels (time point 1) exceeding the cut-off for clinically significant PTSD symptoms were found in control (60.3%) and intervention (53%) groups. After intervention was delivered and symptoms assessed immediately (time point 2), there was no significant reduction in PTSD symptoms in intervention (45.5%) or control groups (56.6%). Results for both groups were merged at time point 3 after the control group received the intervention, and followed up at time point 4 (see Table 2); however, no significant change occurred between time points 3 (47.6%) and 4 (48.8%).

##### Advancing Adolescents

In the study by Panter-Brick et al,^27^ Syrian participants had higher symptom scores than Jordanian participants for all measured outcomes (*P* = 0.016 compared with *P* < 0.001). The intervention was not effective in decreasing post-traumatic stress symptoms.

#### Other clinical psychological outcomes

##### Teaching Recovery Techniques

In the study by Barron et al,^32^ depression levels were the same across intervention and control groups at baseline. There was a significant reduction in depression score in the intervention group at follow-up (*d* = 1.24).

In the second study by Barron et al,^33^ depression levels were the same in intervention and control groups at baseline; however, depression scores were slightly higher in females in the intervention group (female mean: 17.10, s.d. = 4.96) compared with females in control group (female mean: 15.17, s.d. = 4.83). No significant reduction was observed in the intervention group post-intervention (*P* = 0.746). However, the control group experienced an increase in depression scores (increase in mean from 14.71 to 16.2). This increase was significantly related to gender, where authors observed a statistically significant increase in female depression scores in the control group (*P* < 0.01). No differences were found at baseline between intervention and control groups regarding peritraumatic dissociation after completing the intervention.

In the study by El-Khani et al,^35^ a significant reduction in DSRS scores was identified at both time points 2 and 3 for both TRT + P groups (time points 2 and 3: *P* < 0.001) and TRT (time point 2: *P* = 0.003; time point 3: *P* = 0.032), whereas no such change was reported for the waitlist group. Average anxiety scores were high (above the clinical cut-off of 34) in all three groups at baseline, decreasing at time point 2, then increasing at time point 3 in the waitlist group. Although there was a slight increase between time points 2 and 3 in the TRT group, the overall decrease between time points 1 and 3 was significant (*P* < 0.001). The TRT + P group demonstrated the most robust improvement across the three time points (*P* < 0.001). Psychological distress was high in all three groups at baseline, and only decreased significantly at time point 2 in the TRT + P group, before increasing somewhat by time point 3 (no significant changes overall).

In the study by Qouta et al,^34^ there was no evidence of improvement in depressive symptoms or psychological distress at time point 2 (post-intervention) or time point 3 (6-month follow-up). Authors reported a clinically significant increase in distress symptoms (see B.4 Adverse Events section).

##### Writing for Recovery

In the study by Lange-Nielsen et al,^31^ time had an effect on depression symptoms, increasing the score overall (*P* = 0.000). More importantly, there was a statistically significant increase in depression scores in the intervention group, with the percentage scoring above clinical cut-off increasing from 38.5% at time point 1 to 89.4% at time point 2. However, among the merged sample at time points 3 and 4, there was a significant reduction in students scoring above the cut-off of clinical diagnosis between time point 3 (91.1%) and time point 4 (44.3%). There were no significant differences in depression scores between time point 1 (intervention: 38.5%; control: 43.1%) and time point 4 (44.3%). Clinically important anxiety levels did not differ between intervention and control groups at time point 1, neither at time points 2, 3 and 4 (time point 1: intervention 27.3%, control 22.4%; time point 2: intervention 33.3%, control 22.4%; time point 3: merged sample 30.6%; time point 4: merged sample 26%).

##### Advancing Adolescents

In the study by Panter-Brick et al,^27^ improvements in insecurity, distress and mental health difficulties were reported. Using the Human Insecurity and Human Distress Scale, the intervention group showed sustained impact post-intervention (*P* < 0.01). As scores from both participant nationalities (i.e. participants from Jordan and Syria) were grouped together, it was not possible to report on our sample of interest for these outcome measures.

#### Demographics and war and trauma exposure outcomes

##### Age and gender

One study did not report any statistics on age and gender of children participants other than being aged between 9 and 12 years old.^35^ Overall, there was no significant effect of moderating factors such as age and gender on main reported outcomes in other included studies, except for the increase in female depression scores in the control group (*P* < 0.01) in the TRT study by Barron et al.^33^ However, age/gender differences for some interventions were reported at baseline/pre-tests in included studies, and are discussed below.

##### Teaching Recovery Techniques

One TRT study reported gender difference on the amnesia subtest, as females in intervention and waitlist groups had lower level of dissociative amnesia (*P* < 0.01).^33^ In the same study, females in the waitlist group experienced higher levels of war stressor pre-test and post-test (*P* ≤ 0.01). In the second TRT study,^32^ significantly higher levels of traumatic grief were reported among females pre-test. In the third TRT study, significantly more males (22.5%) dropped out than females (9.2%; *P* < 0.001).

##### Advancing Adolescents

In the Advancing Adolescents study, except for prosocial behaviour and post-traumatic stress reactions, females reported higher baseline symptoms than males for all outcomes (*P* < 0.003 to *P* < 0.0001).^27^

### Adverse effects

Two studies reported adverse effects at time point 2; one reported an increase in depression symptoms in the WfR intervention^31^ and another reported a transient increase in the proportion of children with clinically significant psychological distress.^34^

### Coping skills and support

Three studies reported children's and adolescents’ qualitative comments about the effect of the intervention on their social life and well-being.^27,31,32^ In addition to finding a small effect (*d* = 0.35) of reduced impact of trauma on performance at school, students in one of the TRT studies^32^ positively appraised their experience. They reported feeling relaxed and optimistic, and that their social communication, self-awareness and self-responsibility had improved. Students in a second study^31^ reported intervention participation as a positive experience at time point 3 (88%), which increased at time point 4 (94.3%). A third study^27^ reported improvement in psychosocial well-being of participants post-intervention, increased ability to trust others and having made more friends.

### Study quality and risk of bias

We found evidence of high risk of bias and unclear reporting among several studies included in the review (Table 3). Because of the nature of face-to-face psychological intervention research, blinding of teachers, therapists and researchers was often not possible. Authors of three studies noted this specifically: Qouta et al^34^ mentioned blinding without specific detail, Panter-Brick et al^27^ noted that participants and fieldworkers were blinded to group allocation and El-Khani et al^35^ reported that researchers who collected the data were blinded to group allocation. Barron et al and El-Khani et al were the only authors to perform an intention-to-treat analysis.^33,35^

**Table 3:** Risk of bias assessment

	Intervention	Selection bias (random sequence generation)	Selection bias (allocation concealment)	Detection bias	Attrition bias	Reporting bias	Other bias (individually reported)
Barron et al, 2013^32^	TRT	High	Low	Unclear	Low	Low	None
Barron et al, 2016^33^	TRT	Low	Unclear	Unclear	Low	Low	None
Lange-Nielsen et al, 2012^31^	WfR	High	Unclear	Unclear	High	Low	High
Panter-Brick et al, 2018^27^	Advancing Adolescents	Low	Low	Unclear	Low	Low	None
Qouta et al, 2012^34^	TRT	High	Unclear	Unclear	High	Low	None
El-Khani et al, 2021^35^	TRT/TRT + parenting	Unclear	Low	Unclear	Low	Low	None

TRT, Teaching Recovery Techniques; WfR, Writing for Recovery.

#### Selection bias

##### Random sequence generation

All participants were randomised. Authors of three out of six studies used low-risk randomisation methods (i.e. coin toss).^27,32,33^ Two studies used high-risk methods, including use of consecutive numbers to randomise participants^31^ and group assignment based on gender.^34^ The last study was ranked unclear risk as they did not mention the randomisation method.^35^

##### Allocation concealment

Only three studies concealed allocation to intervention and control groups.^27,32,35^

#### Detection bias

Most studies did not address detection bias, except one;^31^ however, the information provided was not detailed enough to evaluate bias risk.

#### Attrition bias

All studies had low attrition rates and detailed expected reasons for drop-out, such as changing school during the study or family moving to new location. Attrition bias risk was identified in two studies; however, because of using high-risk methods to deal with missing data, such as replacing missing data with median^31^ or using an estimation method,^34^ these methods were deemed unsuitable.

#### Reporting bias

All studies were evaluated as having low risk of reporting bias.

#### Other sources of bias

Lange-Nielsen et al^31^ reported a study protocol deviation owing to communication difficulties, which meant the waitlist control group completed just one out of two planned assessments before receiving the intervention.

### Intervention fidelity

One study made no mention of intervention fidelity.^27^ Two studies^32,33^ assessed programme fidelity by using a questionnaire completed by observers (who observed intervention sessions to assess adherence to the programme) and school counsellors. They were asked about adherence to programme guidelines, programme adaptation and counsellors’ presentation skills. In the first study,^32^ counsellors reported a high degree of adherence to the programme (94%), unlike observers (60%). Both agreed on good presentation skills. Programme adaptation reasons were related to supporting students to understand, encouraging them to talk, listening to their experiences and managing session time. In the second study,^33^ counsellors and observers agreed that the guidelines were followed to an acceptable extent, and both agreed on good presentation skills.

Three remaining studies used three methods to evaluate fidelity: for WfR, video recordings showed deviation from the manual, i.e. instructors omitted telling students not to blame themselves;^31^ for one study using TRT, weekly supervisory and preparatory meetings with the teacher and the counsellor were employed as a training technique to improve treatment fidelity;^34^ a second study using TRT provided continued supervision and support via online medial like Skype, WhatsApp and email, in addition to some interviews that were conducted with facilitators.^35^

## Discussion

### Key findings

The aim of this systematic review was to evaluate the effectiveness of available RCT-assessed psychosocial interventions for children and adolescents who have been exposed to war and conflict in LMICs in the Middle East, and subsequently experience PTSD and other psychological disorders symptoms, compared with control groups. Demographic effects, other beneficial outcomes and/or side-effects were also evaluated.

Previous reviews focusing on children and adolescents in LMICs more broadly found that psychological intervention, especially those that are trauma-focused, may effectively treat PTSD^22^ or have beneficial effect on PTSD symptoms and other psychological problems such as depression and anxiety.^8,24^ But our work highlights unique findings based on LMICs in the Middle East. Two out of three of the included interventions originated from trauma-informed interventions and were delivered in school-based settings. TRT was used in four studies in this review^32–35^ and was CBT-oriented. The fifth study intervention (WfR^31^) was narrative therapy derived, and the final study used a group psychosocial intervention focusing on well-being in general (Advancing Adolescents).^27^ We identified three interventions with promising components, but overall weak evidence of effectiveness. One out of six studies carried out follow-up assessments at 4/5 months.^31^ but did not demonstrate any long-term treatment effect. Except for three RCTs evaluating TRT,^32,33,35^ no other studies demonstrated a statistically significant treatment effect.

Overall, because of the small number of available RCTs, and with samples limited to Palestinian children living in Gaza and the West Bank, and Syrian children living in Jordan and Lebanon, it is not possible to make general statements on the effectiveness of psychological interventions for children and adolescents in LMICs in the Middle East in need of support for PTSD and other psychological disorders. Furthermore, dedicating future research to cover this topic in LMICs in the Middle East is urgent and essential.

### Comparison with prior work

Of the three interventions used across six studies, TRT was the only programme that demonstrated a statistically significant reduction in PTSD scores.^32,33,35^ Results are interpreted cautiously, however, as follow-up assessments were carried out just 2 weeks post-intervention in two studies, which was the shortest follow-up period of all included studies. The third study reported significant reduction up to 12 weeks post intervention, with the best improvement happening at the enhanced TRT group with a parenting component.^35^ A lack of long-term effectiveness data has been highlighted by others,^50,51^ and will be addressed by an upcoming RCT evaluating TRT for refugee children from Middle Eastern LMICs.^52^ Other previous reviews^23,24^ did not specify a certain psychosocial intervention, but found suggestive evidence of decreasing PTSD symptoms of group-based and trauma-focused psychosocial interventions. TRT has been evaluated with children displaced by war living in the UK^50^ and Sweden,^51^ where statistically significant reductions in post-traumatic symptoms were reported. Qualitative data indicates that TRT may help to normalise experiences, teach coping techniques (i.e. breath control) and facilitate understanding of experience.^51^ A strength of TRT is that it could be integrated within existing communities and delivered by a trained professional, such as a teacher, with established rapport among students. The TRT programme can consist of as little as five sessions, which is 50% less than the average of other interventions for children and adolescents in LMICs.^22^ Implementation may therefore be less susceptible to barriers caused by ongoing armed conflict, lack of financial resources and attrition.

Depression often co-occurs in people affected by war who have PTSD,^53^ and when we evaluated secondary outcomes, we found this to be the next most commonly assessed variable in five out of six studies. Authors of two studies evaluating TRT reported a significant reduction in depression,^32,35^ consistent with past research^51^ and results of a past systematic review conducted with youth in LMICs.^8^ Aside from this, we also found that participants gained clinically significant psychosocial benefits from two interventions (TRT^32,33^ and WfR^31^), such as improvements in school performance, communication, number of friends, self-awareness and self-responsibility. In settings where children and adolescents may be separated from their parents^33^ and social structures fractured by conflict, support and connectedness are especially important for building self-esteem that can be protective in the face of psychological concerns.^54^ Findings from the Advancing Adolescents study^27^ and one TRT study^35^ indicated that participants felt reduced insecurity, distress and mental health difficulties, which are important for building resilience.^55^ In one TRT study, non-medical factors such as school performance were assessed as a proxy for psychosocial well-being, and it was argued in this study that the TRT intervention allowed children and adolescents to understand educational gains, suggesting an improvement in student perception of learning capacity.^32^ These non-medical elements are important as they reflect another aspect of coping with daily stressors that may be supportive without necessarily resulting in a measurable health effect.

The presence of adverse outcomes in several studies warrants attention. Study authors reported adverse effects following two out of three interventions (TRT^34^ and WfR^31^), including increased distress and depression. Several possible causes may explain these findings. First, timing of data collection is important, particularly as mental health symptoms may fluctuate over time.^56^ Authors of the WfR study^31^ noted an increase in depression scores between the intervention and first follow-up (time point 2). They suggest that the short follow-up interval (19 days) and effect of recalling traumatic memories might have caused this elevation. This is supported by the decrease in depression scores between time points 3 and 4, and the overall non-significant difference between scores for time points 1 and 4. A second explanation is that interventions may have differential effects on individuals and subgroups. For instance, a previous study^57^ found that children from one classroom with high exposure to war appeared to account for the significant difference in depression scores, compared with those with low exposure.^57^ In one study by Barron et al, increased depression scores were only recorded for girls in the control group post-test; however, this might be because of higher exposure to war stressors reported pre-test by girls in the control group.^33^ Others have suggested that in areas where conflict is ongoing, the uncertainty and lack of resolution may be an independent risk factor for trauma responses.^58^

Findings from this review suggest limited effectiveness of psychosocial and psychological interventions consistent with one previous systematic review that included adults and children sample in LMICs;^5^ however, this may also reflect the challenging circumstances under which the research takes place.^59^ War exposure adds complexity to undertaking research,^31,34^ and was high across all included studies. Interventions used with Syrian refugees were implemented in post-war settings, as studies were done in host countries like Jordan and Lebanon;^27,35^ however, conflict was still ongoing in Syria. In the other included studies, intervention implementation or data collection were during an ongoing conflict.^31–33^ Experiences of shelling and bombardment were high across three studies (79–86%), based in Nablus,^33^ Gaza^31^ and Jordan.^27^ For interventions to be feasible in war-affected environments, they must address the impact of ongoing conflict on mental health and everyday life.^56^ Treatment effects may be underestimated^32^ or lost in a situation where violence is reoccurring.^60^ Certain communities or districts can also be affected at different times,^56^ affecting results. Although some argue that offering an intervention post-war may achieve better results,^57^ there is a clear need for interventions that fit the needs of children and adolescents who live and grow up in conflict and war-affected settings.

### Future research

In five out of six studies, authors confirmed that the interventions were culturally accepted and appropriate or suitable with low-resource settings (TRT^32–35^ and Advancing Adolescents^27^). For instance, in one study, trained local researchers reviewed content to ensure culturally sensitive interpretations were adopted.^33^ The same study^33^ considered TRT to be culturally appropriate according to previous research results from school counsellors and adolescents in Palestine.^32^ This is important in research where Western interventions are adapted for non-Western settings, as this may affect intervention acceptability and implementation,^56^ and has been recommended for future research in a previous review^8^ highlighting the importance of culturally appropriate assessment of local needs. For instance, although the success of interventions may be enhanced by a supportive adult or caregiver working to support the child throughout the process, this person may not be available in LMICs,^61^ as was observed in several studies included with the review, either because of the lack of knowledge about psychosocial support or the nature of war settings, where one or both parents might be dead or missing. Additionally, the type of relationship between the child or adolescent and their caregiver may differ in distinct contexts.^61^ Evidence suggests that collaborating with local organisations, consulting local mental health specialists^62^ and understanding the differences in relationship between caregiver and child or adolescent in different cultures^63^ may all help to adapt Western-developed interventions in LMICs.

We observed several methodological factors that could be addressed in future research of this nature. First, all studies used self-report measures without utilising a rigorous, systematic evaluation for clinical diagnoses. We also found frequent unclear reporting of methods that hindered a full quality assessment. None of the included studies demonstrated that their methods were bias free; however, four studies^27,32,33,35^ were evaluated as having the least risk of bias out of the group. Potential for bias was most common when high-risk methods of randomisation were used, based on gender^34^ or use of consecutive numbers^31^ to determine group allocation. Random sequence generation, such as use of a coin toss or computer-based generation methods, can improve methodological rigor and should be considered where possible by future researchers.

All included studies used classroom or group/community-based interventions rather than one-to-one therapy, which is expected because of the lower cost of group interventions and lack of CAP services in LMICs in the Middle East. We found variable implementation and cost-effectiveness data reported across the included studies, which is problematic because this information is essential for those tasked with implementation. Intervention fidelity was assessed in five^31–35^ out of six included studies; however, many of the self-report methods used were subject to bias. Use of videotaping and objective evaluation was employed in one study,^31^ and is considered a more accurate method to assess treatment fidelity;^62^ however, this is not always feasible in war-affected settings.^57^ Cost-effectiveness data were reported in one out of six studies,^33^ calculated at US$38.68 per student participating in TRT. Although this may seem inexpensive, in LMICs where there are shortages in basic needs such as food, water and housing, these are prioritised over psychological interventions and research.^64^ Nevertheless, where possible, researchers should report implementation data such as cost-effectiveness,^62,65^ as this will help guide decision-makers in the context of limited resources and healthcare funding.^66^

El-Khani et al^31^ highlighted their success in implementing the TRT intervention online as a result of the COVID-19 pandemic, and the feasibility of such implementation. They proposed that using technology for online implementation would have potential benefits for settings lacking accessibility to such services, increase opportunities in LMICs,^35^ and prevent further lack of services and marginalisation owing to the COVID-19 pandemic. Further studies are needed to address this point and to understand the effect of COVID-19 on such interventions (which is beyond the scope of this study).

The heterogeneity of included interventions – in terms of target problems and underpinning theoretical orientation – means that inferences from this review must be made with caution. One included intervention was a group of community-based activities to provide psychosocial care in non-clinical sittings. And the two other interventions were trauma-informed, either CBT or narrative therapy oriented. The TRT intervention was described in three studies^32,33,35^ as having trauma-focused components or derived from trauma-focused CBT; however, it might be argued that it is a trauma-informed rather than trauma-focused intervention, because of the trauma-specific skills-based nature of the intervention, and that the trauma-focused components are not sufficient compared with traditional trauma-focused CBT. The WfR intervention required participants to write in detail about traumatic events, but the briefness of the intervention and non-guided narration also means that it is better classified as trauma informed. Although, trauma-informed interventions are recommended for surveillance of trauma, and enhancing resilience in children with adverse childhood experiences,^67^ only trauma-focused interventions are recommended by the National Institute for Health and Care Excellence as a first-line treatment for PTSD.^68^ These points need to be addressed in more detail in future studies comparing effectiveness of trauma-informed versus trauma-focused interventions implemented with war-affected children.

Training and supervision for personnel providing psychosocial interventions are important elements for future research, as the lack of available mental health professionals is a major barrier for intervention implementation in LMICs.^69^ Often facilitators in low- and middle-income settings deliver high psychosocial interventions with little to no mental health background, often referred to as task-sharing or task-shifting.^61^ Despite this, five out of six studies were evaluated as having an appropriate professional to deliver the intervention. Most had either a formal mental health background,^32–34^ were teachers trained specifically to deliver the intervention^25,35^ or used interventions that did not require mental health background.^31^ Although the task-shifting model may be desirable and cost-effective, the availability of supervision and training is still needed for lay counsellors,^62^ non-mental health professionals and families involved with service delivery.^69^ For circumstances where facilitators have minimal mental health training, preliminary research suggests that it may be feasible for remote supervision to be provided,^70^ where specialist mental health services are unavailable locally.

### Strengths and limitations of the review

To our knowledge, this is the first systematic review focusing on interventions for children and adolescents with PTSD in LMICs in the Middle East. We carried out a detailed review of each study and found several key areas for future researchers to build upon, including the need for follow-up and cost-effectiveness data. RCTs are considered to produce the highest-quality evidence, and so were the intended focus of our review. However, as such study designs tend to be labour-intensive and expensive, they are comparatively rare in LMICs. Important findings from other study designs may therefore have been missed by our review parameters. Given the lack of evidence in this area, future reviewers may consider expanding the age range to include young adults and older adolescents, and to include other conflict-related causes of PTSD, such as state-sponsored violence (e.g. police torture and abuse), as some countries in the Middle East do not suffer from ongoing war but there is widespread violence, identity/ethnic-related attacks and other forms of political violence. It is well-known that RCT results are commonly affected by variability in conditions and populations,^71^ and therefore may not always be feasible in settings affected by ongoing conflict.^54^ Challenges in publishing peer-reviewed research from LMICs are identified across many areas of health research^72^ and are not isolated to research on psychological support alone. Studies not published in peer-reviewed journals or English language might have been missed. Inter-rater reliability and kappa statistic about decisions to include studies were not conducted or reported. Finally, we deviated from PROSPERO record by including one study with the age range of 18 years and under (rather than all under 18 years). However, as the average age group for this study was comparable with other included studies, we believe any effect from this adjustment would be negligible, and data was not pooled for quantitative analysis.

In conclusion, this review highlights a paucity of robust evidence on available treatment options for refugee and displaced children and adolescents affected by war from LMICs in the Middle East. Based on the evidence presented, it was not possible to make conclusions regarding the efficacy of the psychosocial interventions for our targeted population, which is also indicative of the broader problem identified from our review, being that there is not enough research in this area. The evidence presented points to a need for continued efforts in developing effective interventions that support children and adolescents affected by war and displacement. Some results are encouraging, as participants reported the acquisition of new social and coping skills, which are needed amid post-war and ongoing conflict, to provide children and adolescents with a sense of safety and stability. Given that several studies reported adverse effects for some interventions, further attention must be given to intervention development and implementation to support this vulnerable population.

## Appendix 1 Detailed Search Methodology

**Table:**

1) OVID search on 3 July 2019 (EMBASE (1974), Medline (1946), Global Health (1973), PsycINFO (1806), (search in Keyword):
2) Effect*
3) Treatment
4) Intervention
5) ‘Psychological intervention’
6) 1 or 2 or 3 or 4
7) PTSD
8) Trauma
9) ‘Post trauma’
10) ‘Post-traumatic stress disorder’
11) 6 or 7 or 8 or 9
12) Child*
13) ‘School age’
14) Adolescent
15) Youth
16) 11 or 12 or 13 or 14
17) ‘Middle east’
18) ‘Arab countries’
19) ‘Middle income’
20) ‘Low income’
21) LMIC
22) 16 or 17 or 18 or 19 or 20
23) War
24) Conflicts
25) ‘Armed conflicts’
26) Refugee
27) ‘Internally displaced’
28) 22 or 23 or 24 or 25 or 26
29) 5 AND 10 AND 15 AND 21 AND 27
30) 5 AND 10 AND 15 AND 21
31) 5 AND 10 AND 15 AND 27
32) PubMed on 3rd of July 2019, search in ‘all fields’: The exact same search with OVID.
Cochrane RCT library search strategy was as the following (All fields search tool was used, on 3 July 2019):
First search:
33) Effect* or Treatment or intervention or ‘Psychological intervention’
And
34) PTSD or trauma or ‘Post trauma’ or ‘Post-traumatic stress disorder’
And
35) Child* or ‘School age’ or Adolescent or Youth
And
36) ‘Middle east’ or ‘Arab countries’ or ‘Middle income’ or ‘Low income’ or LMIC
And
37) War or Conflicts or ‘armed conflicts’ or refugee or ‘internally displaced’
Second search:
1) Effect* or Treatment or intervention or ‘Psychological intervention’
And
2) PTSD or trauma or ‘Post trauma’ or ‘Post-traumatic stress disorder’
And
3) Child* or ‘School age’ or Adolescent or Youth
And
4) ‘Middle east’ or ‘Arab countries’ or ‘Middle income’ or ‘Low income’ or LMIC
Third search:
1) Effect* or Treatment or intervention or ‘Psychological intervention’
And
2) PTSD or trauma or ‘Post trauma’ or ‘Post-traumatic stress disorder’
And
3) Child* or ‘School age’ or Adolescent or Youth
And
4) War or Conflicts or ‘armed conflicts’ or refugee or ‘internally displaced’

## Appendix 2 Excluded Studies

**Table:**

Wrong settings	
Studies in high-income countries in the Middle East:	A Teacher-Delivered Intervention for Adolescents Exposed to Ongoing and Intense Traumatic War-Related Stress: A Quasi-Randomized Controlled Study, Berger et al, 2012. Preventing Children's Posttraumatic Stress After Disaster with Teacher-Based Intervention: A Controlled Study, Wolmer et al, 2011. Helping Youth Immediately Following War Exposure: A Randomized Controlled Trial of a School-Based Intervention Program, Slone et al, 2013. Post-traumatic Reaction of Israeli Jewish and Arab Children Exposed to Rocket Attacks Before and After Teacher-Delivered Intervention. Wolmer et al, 2013. Teacher-Delivered Resilience-Focused Intervention in Schools with Traumatized Children Following the Second Lebanon War, Wolmer et al, 2011. School-Based Intervention for Prevention and Treatment of Elementary-Students’ Terror-Related Distress in Israel: A Quasi-Randomized Controlled Trial, Berger er al, 2007.
Low- and middle-income countries outside the Middle East:	Effectiveness of a School-Based Group Psychotherapy Program for War-Exposed Adolescents: A Randomized Controlled Trial, Layne et al, 2008. Children and Mothers in War: An Outcome Study of a Psychosocial Intervention Program, Dybdahl et al, 2001.
Refugee studies in high-income countries in Europe:	Changes in Traumatic Memories and Posttraumatic Cognitions Associate with PTSD Symptom Improvement in Treatment of Multiply Traumatized Children and Adolescents, Kangaslampi et al, 2019. Changes in Traumatic Memories and Posttraumatic Cognitions Associate with PTSD Symptom Improvement in Treatment of Multiply Traumatized Children and Adolescent, Kangaslampi et al, 2020. Narrative Exposure Therapy for 7- to 16-year-olds: A Randomized Controlled Trial with Traumatized Refugee Children, Schauer et al, 2010. Narrative exposure therapy for immigrant children traumatized by war: study protocol for a randomized controlled trial of effectiveness and mechanisms of change, Kangaslampi et al, 2015. Trauma-focused cognitive behavioral therapy with unaccompanied refugee minors: a case series, Unterhitzenberger et al, 2015. Narrative exposure therapy for PTSD increases top-down processing of aversive stimuli evidence from a randomized controlled treatment trial. Andenauer et al, 2011.
Analysis of an already included study	Psychosocial Group Intervention Among War-Affected Children: An Analysis of Changes in Posttraumatic Cognitions, Kangaslampi et al, 2016.
Wrong population, post-traumatic stress disorder owing to child abuse or mixed sample (children and adults with no distinction in results according to the age)	1) Trauma-focused cognitive behavioral therapy: Cultural adaptations for application in Jordanian culture, Damra et al, 2014. 2) Enhancing Need Satisfaction to Reduce Psychological Distress in Syrian Refugees, Weinstein et al, 2016.
Same sample as an already included study	Hair cortisol concentrations in war-affected adolescents: A prospective intervention trial, Dajani et al, 2018. C-reactive protein, Epstein-Barr virus, and cortisol trajectories in refugee and non-refugee youth: Links with stress, mental health, and cognitive function during a randomized controlled trial, Pantir-Brick et al, 2020. Effectiveness of psychosocial intervention enhancing resilience among war-affected children and the moderating role of family factors, Qouta et al, 2015. The Role of Attachment and Emotion Regulation in the Psychosocial Intervention Among War-Affected Children, Eloranta et al, 2017.
Not a randomised controlled trial	Evaluation of a School-Based, Teacher-Delivered Psychological Intervention Group Program for Trauma-Affected Syrian Refugee Children in Istanbul, Turkey, Gormez et al, 2017. Group Crisis Intervention for Children during Ongoing War Conflict, Thabet et al, 2005. Effectiveness of School-Based Intervention in Enhancing Mental Health and Social Functioning Among War-Affected Children, Peltonen et al, 2012. Effectiveness and Specificity of a Classroom-based Group Intervention in Children and Adolescents Exposed to War in Lebanon, Karam et al, 2008. A Short-Term Approach for Promoting Oral Health of Internally Displaced Children with PTSD: the Key Is Improving Mental Health—Results from a Quasi-Randomized Trial, Hamid et al, 2021.
Not measuring the primary outcome (post-traumatic stress disorder)	Can Psychosocial Intervention Improve Peer and Sibling Relations among War-affected Children? Impact and Mediating Analyses in a Randomized Controlled Trial, Diab et al, 2014. The Impact of Structured Activities among Palestinian Children in a Time of Conflict, Loughry et al, 2006. Caregiving for Children through Conflict and Displacement: a Pilot Study Testing the Feasibility of Delivering and Evaluating a Light Touch Parenting Intervention for Caregivers in the West Bank, El-Khani et al, 2020.
Registered randomised controlled trial (no peer-reviewed published results)	Phone-Delivered Psychological Intervention (t-CETA) for Mental Health Problems in 8–17-Year-Old Syrian Refugee Children, Pluess et al, 2019.
